# Psychiatric diagnoses and punishment for misconduct: the effects of PTSD in combat-deployed Marines

**DOI:** 10.1186/1471-244X-10-88

**Published:** 2010-10-25

**Authors:** Robyn M Highfill-McRoy, Gerald E Larson, Stephanie Booth-Kewley, Cedric F Garland

**Affiliations:** 1 Behavioral Science and Epidemiology Program, Naval Health Research Center, San Diego, California, USA; 2 Department of Family and Preventive Medicine and Moores UCSD Cancer Center, University of California, San Diego, California, USA

## Abstract

**Background:**

Research on Vietnam veterans suggests an association between psychological problems, including posttraumatic stress disorder (PTSD), and misconduct; however, this has rarely been studied in veterans of Operation Iraqi Freedom or Operation Enduring Freedom. The objective of this study was to investigate whether psychological problems were associated with three types of misconduct outcomes (demotions, drug-related discharges, and punitive discharges.)

**Methods:**

A population-based study was conducted on all U.S. Marines who entered the military between October 1, 2001, and September 30, 2006, and deployed outside of the United States before the end of the study period, September 30, 2007. Demographic, psychiatric, deployment, and personnel information was collected from military records. Cox proportional hazards regression analysis was conducted to investigate associations between the independent variables and the three types of misconduct in war-deployed (n = 77 998) and non-war-deployed (n = 13 944) Marines.

**Results:**

Marines in both the war-deployed and non-war-deployed cohorts with a non-PTSD psychiatric diagnosis had an elevated risk for all three misconduct outcomes (hazard ratios ranged from 3.93 to 5.65). PTSD was a significant predictor of drug-related discharges in both the war-deployed and non-war-deployed cohorts. In the war-deployed cohort only, a specific diagnosis of PTSD was associated with an increased risk for both demotions (hazard ratio, 8.60; 95% confidence interval, 6.95 to 10.64) and punitive discharges (HR, 11.06; 95% CI, 8.06 to 15.16).

**Conclusions:**

These results provide evidence of an association between PTSD and behavior problems in Marines deployed to war. Moreover, because misconduct can lead to disqualification for some Veterans Administration benefits, personnel with the most serious manifestations of PTSD may face additional barriers to care.

## Background

Numerous studies have demonstrated that exposure to combat or other traumatic events is associated with an increase in psychiatric problems, including depression, substance abuse, anxiety disorders, and posttraumatic stress disorder (PTSD) [1-3]. Another area of concern is the relationship between combat exposure and antisocial behavior. The media have keenly focused on this topic, as evidenced by the publicity surrounding military misconduct both during and after deployment [4-7].

Research on Vietnam War veterans strongly suggests an association between combat exposure and antisocial and high-risk behaviour [8-11]. Boscarino (1981) found that Vietnam veterans and Vietnam-era veterans had higher levels of drug abuse than non-veterans, after adjusting for demographic factors [8]. Yager, Laufer, and Gallops (1984) found that participation in violence during the Vietnam War was associated with a heightened risk of arrests and convictions, after controlling for pre-service factors [9]. Beckham *et al *(1997) reported that exposure to atrocities during the Vietnam War heightened the risk of engaging in interpersonal violence post-war [10]. Another study found that combat exposure level in Vietnam veterans was associated with post war antisocial behavior, including illegal activities, relationship problems, relationship problems, and reckless driving [11].

Other studies examining the relationship between combat and antisocial behavior have focused on more recent military conflicts [12-15]. Rothberg *et al *(1994) found that U.S. Army units that deployed during the Persian Gulf War had higher rates of drug and alcohol service use than did non-deployed units [12]. The 2005 Department of Defense Survey of Health Related Behaviors found that approximately 16-18% of Marines who served in Operation Iraqi Freedom, Operation Enduring Freedom, or other operations reported illegal drug use during the past year, compared with 9% of those who did not serve in any operation [13]. Killgore *et al *(2008) found that Operation Iraqi Freedom soldiers exposed to violent combat reported more aggressive behaviors following deployment, including angry outbursts, destroying property, and threatening others with violence [14].

It has been proposed that PTSD could mediate the relationship between combat and subsequent antisocial behaviour [16-19]. However, research on this topic has produced conflicting findings. Some studies have found that veterans with combat-related PTSD report higher rates of interpersonal violence, incarcerations, and drug use/dependence, compared with veterans without PTSD [10,20-22]. However, not all studies have identified an association between combat-related PTSD and these outcomes [23-25].

The inconsistent findings may be due to methodological differences in the research. For example, studies have relied on retrospective [10,19,25] and cross-sectional [3,26] study designs, most likely due to the uncommon occurrence of both the risk factor (trauma resulting in a PTSD diagnosis) and the outcome (misconduct). As a result, the temporal order of events usually was not examined. Case definitions were not consistent across studies and were based on a variety of methods, including a positive result on a symptom-based checklist or survey [11,18], an interview-based diagnosis [16,25], or hospitalization for PTSD [19,23]. Combat veterans were often compared with dissimilar control groups, such as non-deployable personnel or non-veterans, who may have different rates of misconduct outcomes. Outcomes differed substantially across studies making it difficult to make comparisons between studies. Lastly, research in this area has generally focused on veterans of the Vietnam and Gulf wars, and only a few studies have examined psychiatric disorders and misconduct in contemporary combatants.

### Objectives

The goal of this study was to use a population-based approach to examine the relationships between combat deployment, psychiatric problems including PTSD, and misconduct outcomes. The objectives of this study were to ascertain and compare incidence rates of three types of misconduct outcomes (demotions, drug-related discharges, and non-drug-related punitive discharges) among two military cohorts (war-deployed and non-war-deployed Marines), and to determine if having a psychiatric diagnosis, including PTSD, was associated with misconduct.

## Methods

### Subjects

A population-based cohort study was conducted among all active-duty, enlisted Marine Corps personnel who first entered the military between October 1, 2001, and September 30, 2006. To be eligible for this study, Marines had to have been enlisted for longer than 6 months and deployed to either Iraq, Afghanistan, or Kuwait (war deployed Marines) or to another location outside of the United States without receiving hazardous duty pay (non-war-deployed Marines) before the end of the study period, September 30, 2007. The analyses were limited to active-duty Marines because medical data were not consistently available for reservists.

Excluded from the study were individuals who served less than 6 months of service, did not deploy before the end of the study period, changed military branches during the study time frame, or received hazardous duty pay but did not deploy to Iraq, Afghanistan, or Kuwait. Officers and warrant officers were excluded because they constituted an extremely small portion of personnel who received a misconduct outcome during this time frame.

This research was conducted in compliance with all applicable federal regulations governing the protection of human subjects in research. The Naval Health Research Center Institutional Review Board approved this study (protocol NHRC.2005.0003).

### Data sources and variables

Personnel, demographic, and deployment information collected from the Defense Manpower Data Center (DMDC) and medical information collected from the TRICARE Management Activity were used to construct the longitudinal database for this study. Demographic and personnel predictors included sex, race (Caucasian, African American, Hispanic, or other), date of military entry, accession age (age at military entry,) and Armed Forces Qualification Test (AFQT) cognitive ability score. AFQT was divided into tertiles based on the distribution of scores (low: 0-50, medium: 51-70, and high: 71-100). Age at military entry was dichotomized based on the mean of the distribution (<19, ≥19 years).

Deployment information included dates and country of deployment. Individuals were categorized as being war deployed if they received a combat zone tax exclusion or hazardous duty/imminent danger pay and were deployed to Iraq, Kuwait, or Afghanistan before the end of the study period (n = 77 998.) Personnel whose duty station was outside of the United States and who did not receive hazardous duty pay were classified in the deployed, non-war-deployed cohort (n = 13 944.)

The three outcomes of the study (demotions, drug-related discharges, and non-drug-related punitive discharges) and the dates of their occurrence were obtained from DMDC. Individuals were classified as demoted if official records indicated a lowering of their paygrade. Individuals were classified as having a drug discharge if they were discharged and their separation code description included drug use or abuse. Individuals were classified as having a non-drug-related punitive discharge if they were discharged and their separation code description included frequent involvement with civil or military authorities, court martial or action in lieu of court martial, or a civil or military conviction. This last outcome measure reflects the most severe instances of blatant criminal conduct. In order to classify individuals into the appropriate deployment cohort, all outcomes included in the analyses had to have occurred after a deployment.

Information on inpatient and outpatient medical visits were obtained from Tricare Management Activity, the Department of Defense's health care system. This database includes treatment dates and clinical diagnoses by credentialed providers (including psychiatrists, psychologists, and medical doctors) at both military treatment facilities and government-reimbursed private providers. These direct care records are generated for military personnel on every medical encounter, with the exception of medical encounters that occurred in a war zone or via civilian providers who were not reimbursed through TRICARE.

Individuals were defined as having a PTSD diagnosis if medical records included an *International Classification of Diseases*, Ninth Revision, Clinical Modification (ICD-9-CM) diagnosis code of 309.81. This definition is based on meeting the criteria stipulated in the Diagnostic and Statistical Manuel of Mental Disorders IV (Text Revision) (DSM-IV-TR). and is consistent irrespective of individual combat experiences [27].

Individuals were defined as having a psychiatric diagnosis (excluding PTSD) if their medical records included an ICD-9-CM diagnosis code in the range of 290 and 316, with the exception of 305.1 (tobacco use disorders), 309.81 (PTSD), and 292 and 305.2 to 305.9 (drug-induced mental disorders and drug abuse). Psychiatric diagnoses were made using standard DSM-IV criteria. Psychiatric diagnoses (including PTSD) that occurred after the misconduct outcome event were not included.

### Statistical analyses

Frequency distributions for each risk factor and outcome were obtained and stratified by deployment cohort. Categorical variables were analyzed using the chi-square test and continuous variables were analyzed using *t*-tests.

Three separate Cox proportional hazards regression models were used to determine associations between the independent variables (deployment cohort, psychiatric diagnosis status, AFQT score, sex, race/ethnicity, and accession age) on time to each misconduct outcome (demotions, drug-related discharges, and non-drug-related punitive discharges). Cox regression is a type of survival analysis that is used for modeling the effects of several independent variables upon the time to a specific event [28]. In our study, the advantage of using Cox regression is that is allows data from all participants to be included in the calculation of the three misconduct models, even though subjects entered and discharged from the military at different time points during the study period. For each service member in the study, the observation period started at time of entry into boot camp and continued until he or she had a misconduct outcome, was discharged from the military, or died. In each analysis, Marines who did not have the outcome before the end of the observation period were right censored (meaning that outcomes occurring after the end of the observation period were considered missing.)

Regression diagnostics were performed, and no substantial collinearities were detected among model variables (all correlations were ≤.20). With the exception of psychological diagnosis status, all risk factors met the proportional hazards assumption. Because the time interval between entering the Marine Corps and receiving a psychiatric or PTSD diagnosis (if applicable) was different for each participant, psychiatric diagnosis status was treated as a segmented time-dependent covariate in the Cox regression. All individuals were classified as having "no diagnosis" at the start of the study and changed to either "psychiatric diagnosis" or "PTSD diagnosis" at the month of their first diagnosis. Once classified as having PTSD, that classification became final until the end of study.

Univariate analyses were performed using Cox proportional hazards regression. All variables that were significant in the univariate analysis (p < 0.05) were entered into a general adjusted Cox regression model. From the general model, a reduced and final model was obtained for each misconduct outcome using a manual, backward, stepwise elimination approach using an alpha cutoff level of ≤0.05.

Analyses included testing for interaction among psychiatric status and deployment cohort using the likelihood ratio test. Because effect modification between deployment cohort and psychiatric status was statistically confirmed in all misconduct models, the three Cox regression models were stratified by deployment cohort. For all analyses, a two-tailed alpha cutoff level of ≤0.05 was considered statistically significant. All analyses were performed using SPSS, version 16.0 (SPSS Inc., Chicago, Illinois, USA).

## Results

Of the 164 764 Marines who first enlisted during the study period, 91 825 fulfilled the study inclusion criteria (table 1). The study population for both the drug-related discharge and punitive discharge models each included 13 944 non-war-deployed and 77 881 war-deployed personnel. The demotions model consisted of 13 721 non-war-deployed and 74 998 war-deployed personnel. The study population for the demotions model was smaller than for the two discharge models because 3106 Marines were demoted before ever deploying, making them ineligible for inclusion in either cohort in the demotions model.

**Table 1:** Demographic Characteristics in Three Groups of Marines Corps Personnel, 2001-2007

Characteristic	Non-war deployed	War deployed	**Excluded from study sample**^**†**^
	
	N (%) n = 13 944	N (%) n = 77 881	N (%) n = 72 939
Accession age			
<19 years	6795 (48.7)	37 698 (48.4)	32 719 (44.9)**
≥19 years	7149 (51.3)	40 183 (51.6)	40 219 (55.1)**
Sex			
Male	12 296 (88.2)	74 962 (96.3)**	65 780 (90.2)**
Female	1648 (11.8)	2919 (3.7)**	7159 (9.8)**
Race/ethnicity			
Caucasian	9050 (64.9)	55 942 (71.8)**	54 191 (74.3)**
African American	1653 (11.9)	5504 (7.1)**	5554 (7.6)**
Hispanic	2171 (15.6)	11 150 (14.3)**	7524 (10.3)**
Other/mixed/missing	1070 (7.7)	5285 (6.8)**	5670 (7.8)
AFQT score			
Low (0-50)	4047 (29.0)	26 409 (33.9)**	21 276 (29.2)**
Medium (51-70)	5006 (35.9)	26 860 (34.5)**	26 291 (36.0)**
High (71-99)	4891 (35.1)	24 612 (31.6)**	24 992 (34.3)**

AFQT, Armed Forces Qualification Test.

^†^Individuals who served <6 months of service, were an officer or a warrant officer, did not deploy before the end of the study period, changed military branches during the study time frame (such as from the Marines to the Army), or received hazardous duty pay but did not deploy to Iraq, Afghanistan, or Kuwait, were not eligible for the study.

*Statistically different from the non-war-deployed reference group (p < 0.05).

**Statistically different from the non-war-deployed reference group (p < 0.01).

Personnel in the war-deployed cohort were significantly more likely to be male, Caucasian, and have a low AFQT score (table 1). Individuals in the war-deployed cohort were significantly more likely to have either no psychiatric diagnosis, or a PTSD diagnosis, while individuals in the non-war-deployed cohort were significantly more likely to have a non-PTSD psychiatric diagnosis (table 2). The incidence of the three misconduct outcomes were higher in Marines deployed outside combat zones than in those deployed to combat zones (table 2).

**Table 2:** Psychiatric and Misconduct Outcomes in War-Deployed and Non-War-Deployed Enlisted Marines Corps Personnel, 2001-2007^†^

Characteristic	Non-war-deployed	War deployed
	
	N (%) n = 13 944	N (%) n = 77 881
Psychiatric diagnosis status		
No diagnosis	11 289 (81.0)	66 577 (85.5)**
Psychiatric diagnosis without PTSD	2584 (18.5)	8979 (11.6)**
PTSD diagnosis	73 (0.5)	2325 (3.0)**
Length of service at first diagnosis		
Mean	20.6	25.6**
SD	12.7	14.9
Misconduct outcomes		
Demotion	1300 (9.7)	4692 (6.5)**
Drug-related discharge	250 (1.8)	1148 (1.5)**
Punitive discharge	184 (1.4)	358 (0.5)**

PTSD, posttraumatic stress disorder.

*Statistically different from the non-war-deployed reference group (p < 0.05).

**Statistically different from the non-war-deployed reference group (p < 0.01).

All independent variables were significant in the univariate analyses (p < 0.05) and were entered into the multivariate models. High AFQT score and female sex were inversely associated with all three misconduct outcomes in both cohorts (tables 3 and 4; see Additional file 1). Compared with personnel with no diagnosis, non-PTSD psychiatric diagnoses were positively associated with all three outcomes. African Americans were at a higher risk for the three misconduct outcomes, with the exception of drug-related discharges among non-war-deployed personnel.

**Table 3:** Multivariate Cox Proportional Hazards Regression Analysis Examining Associations of Psychiatric Diagnosis Status and Drug-Related Discharges in Two Cohorts of Marine Corps Personnel, 2001-2007

	Non-war deployed n = 13 944		War deployed n = 77 881	
	**HR**	**95% CI**	**HR**	**95% CI**

Psychiatric diagnosis status				
No psychiatric diagnosis	1.00		1.00	
Psychiatric diagnosis without PTSD	5.65**	4.37 to 7.29	5.22**	4.59 to 5.94
PTSD diagnosis	5.72**	1.80 to 18.19	8.60**	6.95 to 10.64
AFQT score				
Low (0-50)	1.00		1.00	
Medium (51-70)	0.77	0.59 to 1.02	0.79**	0.69 to 0.90
High (71-99)	0.37**	0.26 to 0.52	0.46**	0.39 to 0.54
Sex				
Male	1.00		1.00	
Female	0.51**	0.33 to 0.77	0.40**	0.24 to 0.55
Race/ethnicity				
Caucasian	1.00		1.00	
African American	0.85	0.59 to 1.23	1.73**	1.46 to 2.05
Hispanic	0.41**	0.26 to 0.65	0.63**	0.52 to 0.77
Other/mixed/missing	0.71	0.42 to 1.18	0.75*	0.57 to 0.98
Accession age				
<19 years	1.00		1.00	
≥19 years	1.01	0.79 to 1.30	0.91	0.81 to 1.02

AFQT, Armed Forces Qualification Test; CI, confidence interval; HR, hazard ratio; PTSD, posttraumatic stress disorder.

*p < 0.05

**p < 0.01

**Table 4:** Multivariate Cox Proportional Hazards Regression Analysis Examining Associations of Psychiatric Diagnosis Status and Punitive Discharges in Two Cohorts of Marine Corps Personnel, 2001-2007

	Non-war deployed n = 13 944		War deployed n = 77 881	
	**HR**	**95% CI**	**HR**	**95% CI**

Psychiatric diagnosis status				
No psychiatric diagnosis	1.00		1.00	
Psychiatric diagnosis without PTSD	5.63**	4.18 to 7.58	5.20**	4.11 to 6.58
PTSD diagnosis	2.88	0.40 to 20.79	11.06**	8.06 to 15.16
AFQT score				
Low (0-50)	1.00*		1.00	
Medium (51-70)	0.76	0.54 to 1.05	0.66**	0.52 to 0.83
High (71-99)	0.48**	0.33 to 0.72	0.45**	0.33 to 0.60
Sex				
Male	1.00		1.00	
Female	0.52**	0.32 to 0.84	0.38**	0.19 to 0.77
Race/ethnicity				
Caucasian	1.00		1.00	
African American	2.29**	1.60 to 3.28	2.45**	1.85 to 3.25
Hispanic	0.99	0.64 to 1.54	1.08	0.80 to 1.45
Other/mixed/missing	1.16	0.66 to 2.02	1.23	0.81 to 1.88
Accession age				
<19 years	1.00		1.00	
≥19 years	1.20	0.90 to 1.61	0.69**	0.56 to 0.85

AFQT, Armed Forces Qualification Test; CI, confidence interval; HR, hazard ratio; PTSD, posttraumatic stress disorder.

*p < 0.05

**p < 0.01

Deployment to war was not associated with an increased risk of a drug-related discharge (table 2). In the non-war-deployed cohort, Marines with PTSD were 5.7 times as likely to have a drug-related discharge compared with Marines without a psychiatric diagnosis, after adjusting for all other covariates in the model (p < 0.01; 95% confidence interval [CI], 1.80 to 18.19) (table 3). In the war-deployed cohort, Marines with PTSD were 8.6 times as likely to have a drug-related discharge compared with Marines without a psychiatric diagnosis, after adjusting for other covariates in the model (p < 0.01; 95% CI, 6.95 to 10.64) (table 3).

General psychiatric diagnoses increased the risk for a punitive discharge in both cohorts, but PTSD diagnoses only increased the risk for a punitive discharge in the war-deployed cohort (table 4). Marines in the war-deployed cohort who had a PTSD diagnosis were 11.1 times more likely to have a misconduct discharge compared with their peers who did not have a psychiatric diagnosis (p < 0.01; 95% CI, 8.06 to 15.16).

In both cohorts, a psychiatric diagnosis was associated with an increased risk of a demotion, after controlling for demographic predictors (in the non-war-deployed cohort hazard ratio, 4.5; 95% CI, 4.03to 5.03; in the war-deployed cohort HR, 3.9; 95% CI, 3.68 to 4.20; see Additional file 1). However, a PTSD diagnosis was only significantly related to a demotion in the war-deployed cohort; individuals with a PTSD diagnosis were 5.8 times more likely to have a demotion compared with Marines without a psychiatric diagnosis.

## Discussion

The main goal of this study was to examine the associations between psychiatric diagnoses, PTSD, and misconduct outcomes among war-deployed and non-war-deployed Marines. The incidence rate of PTSD diagnoses in the war-deployed cohort was 3.0%, which is comparable with other studies among active duty personnel that use diagnoses as inclusion criteria (as opposed to PTSD symptom checklists.) [29]. This study found that for both cohorts, Marines with a non-PTSD psychiatric diagnosis had an elevated risk for all three misconduct outcomes (demotions, drug-related discharges, and non-drug-related punitive discharges). A specific diagnosis of PTSD was also associated with an increased risk for all three misconduct outcomes, but only in the war-deployed cohort. In the non-war-deployed cohort, PTSD was a significant predictor in only one of the three misconduct outcomes (drug-related discharges).

The finding that PTSD increased the risk of drug-related discharges for all Marines is consistent with other literature, and a number of theories have been posited to explain the relationship, including the self-medication hypothesis, the sensation-seeking hypothesis, and the susceptibility hypothesis [25,30,31]. Individuals with comorbid PTSD and substance abuse problems are at an increased risk for interpersonal violence, imprisonment, and homelessness [32-34]. Therefore, our results provide more evidence for the importance of drug abuse screening and counseling among service members with PTSD.

Our study also revealed that PTSD increased the risk for demotions and punitive discharges in war deployers only. One possible explanation for this finding is that war deployers may have relatively higher levels of PTSD symptoms. This explanation would be consistent with a recent finding that military veterans with combat-related PTSD reported more severe symptoms on the Trauma Symptom Inventory than did crime victims with PTSD [35]. Data from the National Vietnam Veterans Readjustment Study showed that specific types of combat exposure were associated with higher PTSD scores [36]. For example, PTSD scores were significantly higher for those who said they had killed compared with those who had said they had not killed [36].

Beckham *et al *(1998) also found that exposure to atrocities was associated with higher PTSD symptom levels, even after controlling for combat exposure [26]. Iversen *et al *(2008) found that United Kingdom military personnel deployed to Iraq who felt their life had been threatened were significantly more likely to have high levels of PTSD symptoms compared with personnel who did not feel their life had been threatened [37]. These findings suggest that psychological and behavioral responses to trauma may be specific to the type of trauma experienced. Compared with other types of traumas, the experience of combat has also been shown to be related to both distinct PTSD symptom profiles and increased aggressive behaviour [10,14,36,38,39], both of which could explain the increased behavioral problems in the war-deployed cohort.

The finding of greatest concern in this study is that combat deployed Marines with a PTSD diagnosis were over 11 times more likely to engage in the most serious forms of misconduct than were combat deployed Marines without a psychiatric diagnosis. This finding is similar to results by Noonan and Mumola (2007), who found that compared with other prisoners, military veterans in prison were less likely to report mental health problems but were more likely to be incarcerated for violent offenses than were other prisoners [40]. In another study of veterans who deployed to the first Gulf War (August 1990 to February 1991), Black *et al *(2005) found that incarcerated veterans were 3.6 times more likely to report PTSD symptoms than were non-incarcerated veterans [20]. Future research should examine the reasons that combat veterans with PTSD are at a higher risk for serious misconduct problems and develop interventions to reduce behavioral problems. Such research is critical, because serious misconduct may lead to disqualification for some Veterans Administration benefits. In addition, personnel with the most serious manifestations of PTSD may face additional barriers to care.

Some military studies examining Navy personnel have found that African Americans have higher rates of involvement in the military's discipline system compared to Caucasians [41-44]. Our study replicated this finding and identified that African Americans in the war-deployed cohort were at an increased risk for all three outcomes compared with Caucasians. In addition, African Americans in the non-war-deployed cohort were also at an increased risk of two types of misconduct: punitive discharges and demotions. More research is required to explore possible factors that moderate this relationship, such as previous trauma exposure, socio-economic status, and military occupation.

The interpretation of these findings is limited by multiple factors. First, cases were identified from service utilization records and were restricted to treatment seeking individuals who had a psychiatric or PTSD diagnosis, and it is likely that additional personnel had symptoms without an official clinical diagnosis. Also, combat deployers are likely made aware of and encouraged to seek psychological care if they are experiencing symptoms at a higher rate than non-deployed personnel. Our study only included misconduct outcomes that were measurable in personnel records, so the relationship between PTSD and undocumented types of misconduct remains unclear. Only Marines were included in the study, so the findings may not generalize to other military populations. Also, subjects only contributed time to our study while they were on active duty. As a result, questions remain about misconduct in veterans who have left the service. Lastly, PTSD was a relatively uncommon event in the non-war-deployed cohort, and this may have made it more difficult to detect significant associations.

## Conclusions

Overall, the results of this study confirm that combat veterans with PTSD and other psychiatric diagnoses have an elevated risk of misconduct outcomes after diagnosis. In addition to treating psychiatric symptoms, mental health treatment providers should address the effect PTSD has on behavioral problems among military personnel deployed to war.

## Competing interests

The authors declare that they have no competing interests.

## Authors' contributions

RMH assisted in developing study design, performed the data analysis, and drafted the manuscript. GEL conceived of the study, developed the study design, and assisted in drafting the manuscript. SBK participated in the data analysis and interpretation, and helped to draft the manuscript. CFG consulted on the study methodology, interpreted the data, and made extensive revisions to the manuscript. All authors read and approved the final manuscript.

## Pre-publication history

The pre-publication history for this paper can be accessed here:

http://www.biomedcentral.com/1471-244X/10/88/prepub

## Supplementary Material

Additional file 1 **Psychiatric Diagnosis Status and Demotions in Deployed and Non-War Deployed Marines**. Multivariate Cox Proportional Hazards Regression Analysis Examining Associations of Psychiatric Diagnosis Status and Demotions in Two Cohorts of Marine Corps Personnel, 2001-2007. Click here for file
