# Supplementary material for: Psychiatric diagnoses and punishment for misconduct: the effects of PTSD in combat-deployed Marines
Source: BMC Psychiatry. 2010 Oct 25;10:88. doi: 10.1186/1471-244X-10-88 (PMC3020681; doi:10.1186/1471-244X-10-88)
Supplement: Additional file 1 — Psychiatric Diagnosis Status and Demotions in Deployed and Non-War Deployed Marines. Multivariate Cox Proportional Hazards Regression Analysis Examining Associations of Psychiatric Diagnosis Status and Demotions in Two Cohorts of Marine Corps Personnel, 2001-2007. [file 1471-244X-10-88-S1.DOC]

**Additional Files**

**Supplemental Table 1 - Multivariate Cox Proportional Hazards Regression Analysis Examining Associations of Psychiatric Diagnosis Status and Demotions in Two Cohorts of Marine Corps Personnel, 2001–2007**

|  | Non-war deployed  n = 13 721 | | | War deployed  n = 74 998 | |
| --- | --- | --- | --- | --- | --- |
|  | HR | 95% CI | HR | | 95% CI |
| Psychiatric diagnosis status |  |  |  | |  |
| No psychiatric diagnosis | 1.00 |  | 1.00 | |  |
| Psychiatric diagnosis without PTSD | 4.51^**^ | 4.03 to 5.03 | 3.93^**^ | | 3.68 to 4.20 |
| PTSD diagnosis | 1.66 | 0.53 to 5.16 | 5.81^**^ | | 5.12 to 6.59 |
| AFQT score |  |  |  | |  |
| Low (0–50) | 1.00 |  | 1.00 | |  |
| Medium (51–70) | 0.82^**^ | 0.73 to 0.92 | 0.79^**^ | | 0.75 to 0.84 |
| High (71–99) | 0.57^**^ | 0.50 to 0.65 | 0.55^**^ | | 0.51 to 0.59 |
| Sex |  |  |  | |  |
| Male | 1.00 |  | 1.00 | |  |
| Female | 0.43^**^ | 0.35 to 0.50 | 0.55^**^ | | 0.47 to 0.65 |
| Race/ethnicity |  |  |  | |  |
| Caucasian | 1.00 |  | 1.00 | |  |
| African American | 1.37^**^ | 1.19 to 1.58 | 1.46^**^ | | 1.33 to 1.59 |
| Hispanic | 0.81^**^ | 0.70 to 0.94 | 0.98 | | 0.91 to 1.06 |
| Other/mixed/missing | 0.96 | 0.79 to 1.16 | 0.94 | | 0.83 to 1.05 |
| Accession age (years) |  |  |  | |  |
| Younger (<19) | 1.00 |  | 1.00 | |  |
| Older (19 and older) | 0.85^**^ | 0.77 to 0.93 | 0.86^**^ | | 0.82 to 0.92 |

AFQT, Armed Forces Qualification Test; CI, confidence interval; HR, hazard ratio; PTSD, posttraumatic stress disorder.

^*^p < 0.05

^**^p < 0.01
